# Direct automatic determination of the methanol content in red wines based on the temperature effect of the KMnO_4_/K_2_S_2_O_5_/fuchsin sodium sulfite reaction system

**DOI:** 10.1039/c8ra00307f

**Published:** 2018-02-23

**Authors:** Yong-Sheng Li, La-Mei Mo, Xiu-Feng Gao

**Affiliations:** School of Chemical Engineering, Sichuan University Chengdu 6100651 China lysgxf2005@qq.com; West China School of Basic Medical Sciences & Forensic Medicine, Sichuan University Chengdu 610065 China xiufengg@163.com

## Abstract

The standard method for methanol assay in wine is based on a methanol/KMnO_4_/H_2_C_2_O_4_/fuchsin sodium sulfite (FSS) reaction system. However, it is difficult to control the degree of colour and the temperature of the reaction product in this assay, and its repeatability is also poor due to the generation of CO and CO_2_ in the reaction. Therefore, to solve these problems, potassium metabisulfite was selected to replace H_2_C_2_O_4_, and an automatic analysis method was developed which can realize rapid and accurate determination of methanol and can be used to make an online analyzer. It was discovered that the reactions of methanol/KMnO_4_ and acetaldehyde/FSS are exothermic, while the reactions of methanol/KMnO_4_ and formaldehyde/FSS are endothermic. Consequently, based on the temperature effect, not only was the interference of ethanol eliminated in detecting methanol in wines, the purpose of the research was achieved to directly and accurately determine methanol without sample pretreatment. By optimizing the system, the obtained conditions for determining methanol in wines were as follows: 20 g L^−1^ concentration for KMnO_4_; 3 g L^−1^ concentration for FSS; 40 cm length for the first reaction coil (RC_1_); 100 cm length for RC_2_; 700 cm (I.D.: 0.8 mm) length for RC_3_; 50 °C for RC_3_; about 20 °C for RC_1_ and RC_2_; 330 μL for the sample volume. The method showed a linear response in the range 25–1000 mg L^−1^, with a 0.6% RSD, 8.8 mg L^−1^ detection limit and 25 samples per h, and was successfully used for testing representative wine samples. It also obtained better accuracy than previous methods. Due to its superiority in automated operation, reproducibility, analysis speed and test cost, this method and system can serve as a supplementary standard for methanol assay, and for the quality control of the winemaking process and the final wine-product, as well as for low-alcohol drinks.

## Abstract 2

We found that methanol/KMnO_4_ and acetaldehyde/FSS are exothermic, formaldehyde/FSS is endothermic, so based on the temperature effect, eliminated ethanol interference in detecting methanol in wines, also realized automatic analysis for methanol without sample pretreatment.

## Graphical abstract

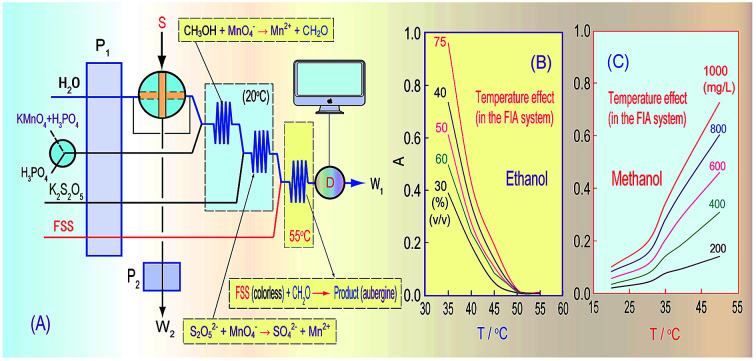

## Introduction

Methanol in wines mainly comes from the hydrolysis of pectic substances^1^ in raw materials and from the deamination of amino acids. The growth of mildew on fermentation materials also produces methanol. In order to improve grape juice yield, pectinase is often added in the fermentation process of wine, but this makes the carboxyl methyl ester in grapes further degrade to methanol.^2^ Methanol in the human body may cause headaches, nausea, blurred vision and so on.^3^ In addition, methanol can cause serious metabolic poisoning of the body.^4,5^ Therefore, the content of methanol during fermented-drink production must be strictly controlled.^6^ Regulations^7^ require that the methanol content in red wine is less than 400 mg L^−1^, where the ethanol content is controlled in the range 9.5–15%. Wine is also recognized as a good drink,^8^ and its consumption significantly increases with humans in improved living standards. Therefore, in order to ensure our health, the determination of the methanol content in wine is very important.

Currently, the methods for determining the methanol content in wine consist of Fourier-transform infrared spectroscopy,^9^ gas chromatography (GC),^10–13^ enzyme-electrode methods,^14,15^ high performance liquid chromatography (HPLC),^16^ fluorimetry,^17^*etc.*

Garrigues *et al.*^9^ researched the simultaneous determination of methanol and ethanol amounts in wine. In their method, methanol and ethanol in wine were firstly volatilized out by heating and then introduced into the infrared spectrometer, but the method was not sensitive enough for the determined target.

A reported enzyme-electrode method^14^ applied a certain electric current onto a platinum electrode which acted by modifying alcohol oxidase (AOD). Alcohols were electrolyzed to form H_2_O under the AOD catalysis. The current change on the electrode in the process was used for the indirect quantitation of alcohols. Although this method was high in sensitivity and selectivity, foreign substances affected the electric current signal from the alcohols. Besides this, Kuo *et al.*^17^ reported a HPLC method for determining methanol in wine, in which the wine samples needed a complex pre-treatment process which resulted in complication of the analysis process and an increase in analysis time.

The flow-injection analysis (FIA)^18,19^ has the advantages of requiring a low dosage of reagent and sample (20–200 μL), having a fast analysis speed (30–300 sample/time), having good reproducibility (RSD < 1.5%), and being able to combine with various detectors^20–27^ or enzyme reactors^28,29^ to make the manual analysis change into automatic analysis. Therefore, it has been widely applied in various analysis and monitoring fields. Fluorimetry based on FIA for methanol was reported once,^30^ in which the authors utilized an immobilized enzyme reactor which was made of alcohol oxidase/catalase (AOD/CAT) and a formaldehyde dehydrogenase (FDH). The method principle was that under catalysis of AOD and CAT, methanol is oxidized by dissolved oxygen to formaldehyde and H_2_O_2_, following which the formed H_2_O_2_ again participated in oxidization of methanol into formaldehyde, and then in catalysis of FDH. The created formaldehyde is further oxidized by NAD^+^ to formic acid and NADH which has a fluorescence feature, and finally, the methanol content is indirectly quantified by the fluorescence intensity of NADH (*λ*_ex_ = 340 nm; *λ*_em_ = 460 nm). However, the method was not used to determine the methanol content in actual red wines or distilled spirits.

Based on the catalytic reaction of AOD and POD, as well as the reaction of H_2_O_2_ with 4-amidopyrine/phenol to generate a coloured product (470 nm), Almuzara *et al.*^31^ reported a stop-flow reversed-FIA system to determine methanol in *Pichia pastoris* fermentation that was producing a heterologous protein, but it also was not used for determining methanol in red wines. Besides, as these methods used free liquid enzymes, this would lead to an increase in consumption of expensive enzymes and test cost.

At present, the test methods for methanol in wine are mainly gas chromatography^32^ and manual colorimetry^33^ based on fuchsin sodium sulfite (FSS),^34^ and the latter is the most commonly used. However, it has some disadvantages, namely, a complicated operation, an unstable colour development and poor reproducibility, as well as being time-consuming.

The analytical principle for the FSS reagent is that methanol in acid medium is oxidized by KMnO_4_ to create formaldehyde. The redundant KMnO_4_ in the reaction system is reduced by adding oxalic acid, and finally, the formed formaldehyde conducts the colour reaction with FSS to create a coloured product. Some research also attempted to improve the colour reaction. Ethyl acetoacetate,^35^ chromotropic acid^36^ and phloroglucin^37^ were once used as the colour reagent, but the effect still was not ideal. Also, using formaldehyde to catalyze the reaction between crystal violet and potassium bromate to analyze methanol was attempted,^38,39^ but the anionic interference was large, so it was not successful in determining the methanol content in wine.

If using GC, before introducing them into the analytical instrument, the wine samples must first be distilled,^40^ which will be time-consuming and cost-increasing in tests. Therefore, it is not suitable for the monitoring of the fermentation process and wine production lines, popularization and application at the grass-roots units. Therefore, this research was conducted to solve the related problems.

## Experimental

### Reagents and instruments

The main reagents which were used in the research were alkaline fuchsin (C_20_H_19_N_3_, Tianjin Damao), methanol, Na_2_SO_3_, concentrated HCl, H_2_SO_4_ and H_3_PO_4_, KMnO_4_ (Chengdu, Kelon), and potassium metabisulphite (Tianjin, Zhiyuan). The water used in the experiment was ultra-purified water (conductivity: 0.065 μS cm^−1^) and all the reagents used were analytically pure.

The following wine samples were purchased: Changyu Cabernet, Ruby Cabernet (China Great Wall, production license number: QS370615020101, QS1300315020006), Muscat, Cabernet Sauvignon (Qingdao, Daze, Yinjia wine factory, production license number: 370215021229, 370215021229), and Gorelli (Georgia).

The analytical instruments and equipment used in the research were as follows: a FIA-3110 type flow-injection processor, a UV-1800PC spectrophotometer with a flow-through cell (Shanghai, Mapada), an AUW120D-type electronic balance (Shimadzu, Japan), an ASB-200 type thermostat (Japan, Jasco), an Aike KL-UP-IV-10 type purified-water device (Chengdu, Kangning), an SC-3000B-011ST type GC (Chongqing, Chuanyi) with a hydrogen flame detector, and a CH-1 type high pure hydrogen generator (Wuhan, Kelin Pufeng).

### Reagent preparation

Methanol storage solution (1.0 g L^−1^): 630 μL of pure methanol solution was added to a 500 mL volumetric flask and diluted to the marker with water. The solution was then stored in a brown reagent bottle with a cover.

Methanol standard solutions: 20, 40, 60 and 80 mL of the methanol stock solution were separately added to 100 mL volumetric flasks and diluted to the required volume with water. The obtained methanol standards were respectively 0.2, 0.4, 0.6 and 0.8 g L^−1^.

Methanol oxidation reagent: 10 g of KMnO_4_ was dissolved in 250 mL of 0.6 mol L^−1^ H_2_SO_4_, into which, 250 mL of H_3_PO_4_ (30%, v/v) was also added. The mixed liquid was stored in a brown bottle.

Fading reagent for KMnO_4_: 40 g of potassium metabisulphite was dissolved in a beaker and diluted to 500 mL with water.

Chromogenic reagent (Schiff reagent): 1.5 g of fuchsin was dissolved in a beaker with 400 mL of water that was at about 80 °C. 15 g of Na_2_SO_3_ was added after the fuchsin solution was cooled. Subsequently, the mixture solution was filtered to obtain its filtrate, and 30 mL of 1.0 mol L^−1^ HCl was again added in the filtrate which was diluted to 500 mL with water. After leaving for 24 h in the dark and adding 0.25–0.5 g of activated carbon, the latter mixed solution was shaken and filtered again. Finally, a colourless reaction reagent (C_20_H_19_N_3_S_3_O_7_HCl) was obtained. Before using, the solution, which was preserved in a 4 °C refrigerator, must return to room temperature.

### Determination principle for methanol

**Fig. 1:**
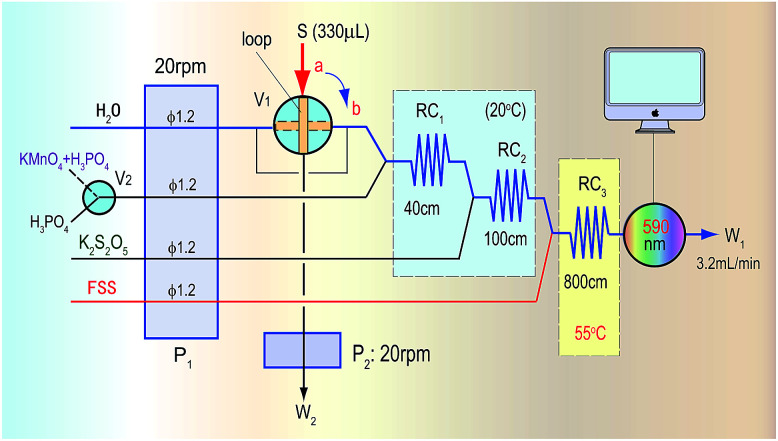
The automatic analysis flow diagram for determination of methanol based on the KMnO_4_/K_2_S_2_O_5_/FSS reaction system and FIA.

Methanol in acid medium is first oxidized by KMnO_4_ into formaldehyde. The formaldehyde then reacts with FSS to form a colourless transitional product that then continues to react with FSS to become a coloured product of blue-violet quinoid (590 nm). Finally, the methanol content in wines is indirectly quantified by detecting the absorbance of the coloured product. The proposed automatic analysis system in Fig. 1 involves the following reaction steps.

In the presence of phosphoric acid, the sample containing methanol (CH_3_OH) was injected into the system, where it quickly reacted with KMnO_4_ to form formaldehyde (CH_2_O):

1 5CH_3_OH + 2KMnO_4_ + 4H_3_PO_4_ → 2MnHPO_4_ + 5CH_2_O + 2KH_2_PO_4_ + 8H_2_O

When the redundant KMnO_4_ met the K_2_S_2_O_5_ that was continuously introduced into the system, the colour of KMnO_4_ itself faded. This avoided affecting the subsequent colour-producing reaction:

2 12KMnO_4_ + 15K_2_S_2_O_5_ + 2H_3_PO_4_ → 12MnSO_4_ + 18K_2_SO_4_ + 2K_3_PO_4_ + 3H_2_O

The formaldehyde formed in the previous step reacted with FSS (C_20_H_21_N_3_S_3_O_7_Cl) to form the final aubergine coloured product (C_22_H_24_N_3_S_2_O_6_Cl), but this reaction is slow and needs to be heated up:

3 2HCHO + C_20_H_21_N_3_S_3_O_7_Cl → C_22_H_24_N_3_S_2_O_6_Cl + HSO_3_^−^

Finally, the methanol content is indirectly quantified according to the absorbance of the coloured product.

### Analysis system and operation processes

When the sampling valve (V_1_) in Fig. 1 was in the load position (the a-position in Fig. 1), the second pump (P_2_) started to take up the sample containing methanol into the loop and to conduct quantitative sampling, during which the redundant sample was discarded (W_2_). At the same time, the mixing reagent of KMnO_4_/H_3_PO_4_ used for oxidizing methanol by a switch valve (V_2_), K_2_S_2_O_5_ and the FSS solution were pushed by the first pump (P_1_) into the first reaction coil (RC_1_), the second reaction coil (RC_2_) and the third reaction coil (RC_3_) in the system, respectively, where these reagents were mixed and could react. Finally, the mixture was pushed into a flow-through photometric detector to obtain a stable baseline signal, which was used as the reagent blank.

When the sampling valve was switched to the injection position (the b-position in Fig. 1), a sample plug containing methanol within the loop was pushed by the water carrier into RC_1_, where the mixing reagent of KMnO_4_/H_3_PO_4_ reacted with methanol to generate formaldehyde. Subsequently in RC_2_ the redundant KMnO_4_ was faded by K_2_S_2_O_5_ to avoid the colour of KMnO_4_ affecting the absorbance of the coloured product that was formed in the final reaction. Then, the formaldehyde generated in RC_1_ flowed through RC_2_ and merged with FSS in RC_3_ to form the coloured product, which was detected as it flowed into the detector at 590 nm. Finally, the methanol content in the sample was determined according to the absorbance value of the coloured product. In determining the methanol content of real red wines, to avoid interference from the original colour of the wine, only the H_3_PO_4_ solution was introduced by the switch valve (V_2_) into the system to obtain a blank absorbance related to the reagent and samples. The methanol content in the sample was then determined by using the absorbance difference of the coloured product.

## Results and discussion

### Examination of reducing agent types

**Fig. 2:**
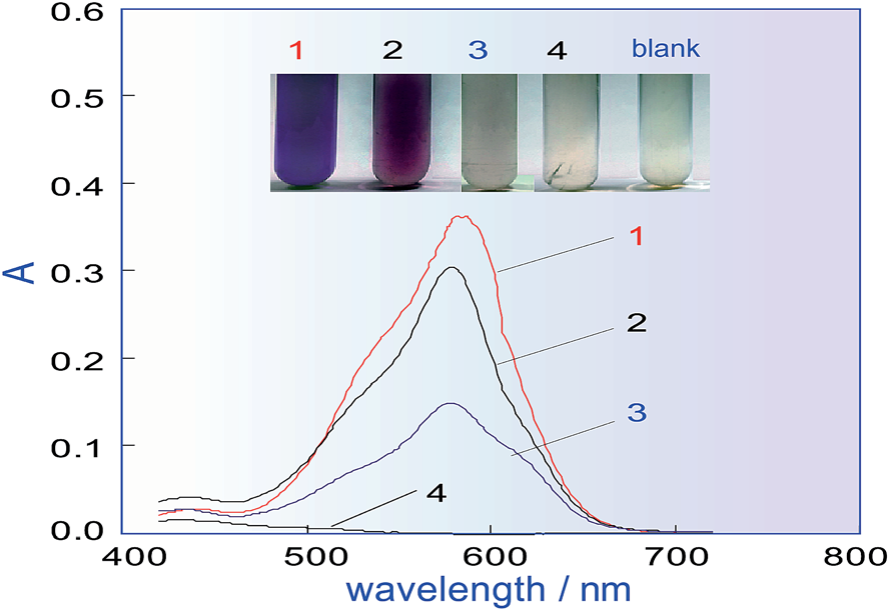
Effects of different reductants on the coloured product. No. 1, 2, 3 and 4 are response curves of the coloured product obtained by respectively using H_2_C_2_O_4_, K_2_S_2_O_5_, Na_2_SO_3_ and AsA as the reducing agent.

In the manual chromogenic method,^33^ H_2_C_2_O_4_ must be used to reduce the excess KMnO_4_ after KMnO_4_ has oxidized methanol to formaldehyde, during which CO and CO_2_ gas are inevitably generated. However, if there is gas in the system shown in Fig. 1, this will seriously affect the system repeatability, so a different reductant must be selected to replace H_2_C_2_O_4_. Some frequently used reductants, like ascorbic acid (AsA), Na_2_SO_3_, K_2_S_2_O_5_, *etc.* were investigated for their ability to fade the KMnO_4_ colour and for factors influencing the methanol determination. A comparison was conducted, for which a 500 mg L^−1^ methanol standard was used as the test sample. The results in Fig. 2 show that when Na_2_SO_3_ or AsA was used as the reductant, absorbance of the coloured product (which relates to the methanol content) was the lowest, and if K_2_S_2_O_5_ was used as the reductant, the absorbance was second only to that of H_2_C_2_O_4_. The biggest advantage of K_2_S_2_O_5_ was that it did not produce CO_2_ gas in the reaction, which is beneficial for improving the repeatability of the process. Therefore, K_2_S_2_O_5_ was chosen as the reductant for methanol assay.

### Orthogonal experiment of the main influencing factors

The H_3_PO_4_ concentration in the KMnO_4_ solution was set at 15% according to the literature,^24^ and the K_2_S_2_O_5_ concentration was set at 80 g L^−1^ before the orthogonal test. The initial parameters of the analytical system were fixed as follows: the revolution speed for P_1_ was 20 rpm, the RC_2_ length was 100 cm (I.D. 0.8 mm) and the sampling volume was 300 μL. Then the orthogonal experiment was conducted. The tested factors (level) were the KMnO_4_ concentration (5, 10, 15, 20 and 30 g L^−1^), the RC_1_ length (60, 100, 150, 200 and 250 cm), the RC_3_ length (200, 300, 400, 500 and 600 cm) and the FSS concentration (0.5, 1.0, 1.5, 2.0 and 3.0 g L^−1^). The FIA detection was conducted using a 1.0 g L^−1^ solution of methanol standard for the test samples at room temperature (25 °C). The obtained results showed that the order of impact on the methanol determination was KMnO_4_ concentration > FSS concentration > RC_3_ length > RC_1_ length. In the preliminary orthogonal experiment, when KMnO_4_ was 20 g L^−1^, FSS was 3 g L^−1^, RC_1_ length was 60 cm and RC_3_ length was 600 cm (0.8 mm I.D.), the response value of the colour-producing reaction relating to the methanol determination was good. However, to get the best results, further optimization was needed.

### Effects of reagent concentrations

#### Optimization of KMnO_4_ concentration

**Fig. 3:**
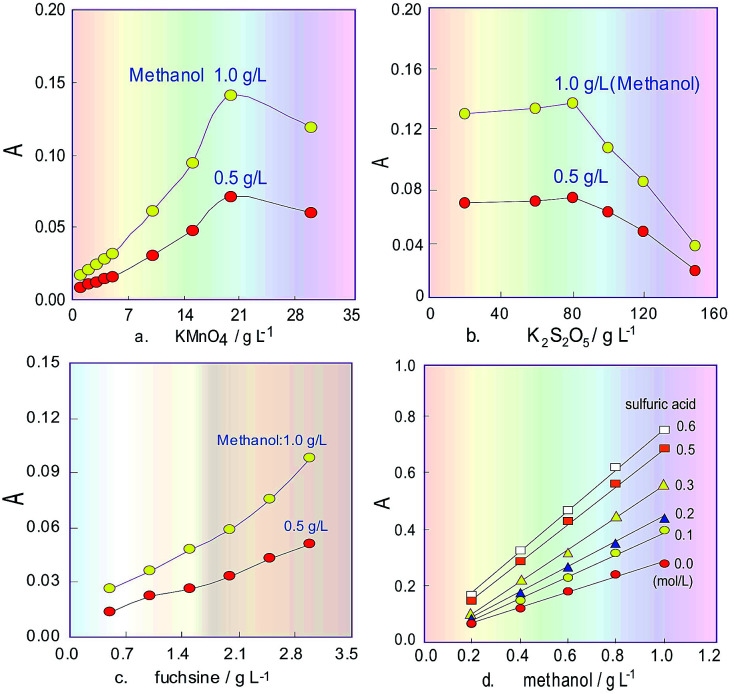
Effects of different reagent concentrations on absorbance of the coloured product. (a) Confirmation of the optimized concentration for KMnO_4_ in the FIA system; (b) optimization of the K_2_S_2_O_5_ concentration; (c) influence of the fuchsin concentration in FSS; (d) effect of the H_2_SO_4_ concentration in the system.

In the new reaction system of methanol/KMnO_4_/K_2_S_2_O_5_/FSS, in order to guarantee the method sensitivity, the KMnO_4_ oxidant must first completely oxidize the methanol in the wine samples into formaldehyde. Therefore in this test, reconfirmation of the orthogonal experiment results was carried out using single-factor optimization experiments. The obtained results in Fig. 3a show that the optimal KMnO_4_ concentration was 20 g L^−1^, which was the same as that in the orthogonal experiments.

#### Optimization of K_2_S_2_O_5_ concentration

K_2_S_2_O_5_ was used to fade the excess KMnO_4_ in the system. Therefore, the K_2_S_2_O_5_ concentration was investigated. The experiment results shown in Fig. 3b indicate that absorbance of the coloured product began to rapidly decrease as the K_2_S_2_O_5_ concentration increased above 80 g L^−1^. The reason for this is that when the concentration was too low, the colour of the remaining KMnO_4_ could not be completely removed, which resulted in the blank increasing the response signal, but when the concentration was too high, the superfluous K_2_S_2_O_5_ introduced in the FIA system would influence the reaction between FSS and the created formaldehyde to lead to a decrease of the coloured product. Therefore, the K_2_S_2_O_5_ concentration was selected as 80 g L^−1^ for the following experiment.

#### Influence of sodium sulfite and fuchsin concentrations on FSS

When FSS and formaldehyde react in the chromogenic reaction, the fuchsin concentration in FSS will directly affect the sensitivity of the FIA system, so the fuchsin concentration was further investigated in the range 0.5–3.5 g L^−1^. In addition to 30 g L^−1^ sodium sulphite in FSS and 80 g L^−1^ K_2_S_2_O_5_ being used, the other testing conditions were kept the same as those mentioned above. The obtained results in Fig. 3c show that the product absorbance associated with methanol linearly increased when the fuchsin concentration was increased. After comprehensively considering the solubility and stability of the fuchsin reagent, its concentration was chosen ultimately to be 3 g L^−1^.

Subsequently, on this basis, the sodium sulphite concentration in FSS was investigated in the range 10–50 g L^−1^. The experiment results showed that if the concentration increased, the product absorbance sharply decreased, and when the concentration was more than 30 g L^−1^, decreasing of the absorbance slowed down. Therefore, the sodium sulphite concentration in the chromogenic agent was selected as 30 g L^−1^.

### Effect of the system acidity

Commonly, H_3_PO_4_ is only used to adjust the acidity of the KMnO_4_ solution, but due to its disadvantages of high viscosity and a tendency to produce bubbles, its concentration cannot be too high in our flow system. Besides, suitable acidity can improve the oxidizability of the KMnO_4_ solution. Therefore, H_2_SO_4_ was added into 15% H_3_PO_4_ solution^24^ as an oxidizing reagent, and its effect on the sensitivity of the system for methanol content analysis was investigated. The obtained results in Fig. 3d show that the absorbance change for the product increased when the H_2_SO_4_ concentration increased, and the slope of the response curves indicates that increasing the H_2_SO_4_ concentration can improve the sensitivity of the system for determining methanol content. However, in the experiment process it was also found that the baseline of the FIA system would drift as the H_2_SO_4_ concentration increased above 0.5 mol L^−1^. Therefore, after comprehensive consideration, the H_2_SO_4_ concentration was selected as 0.3 mol L^−1^. The final oxidant solution consists of 20 g L^−1^ KMnO_4_ and 0.3 mol L^−1^ H_2_SO_4_ as well as 15% H_3_PO_4_.

### Influence of temperature, flowrate and coil length

**Fig. 4:**
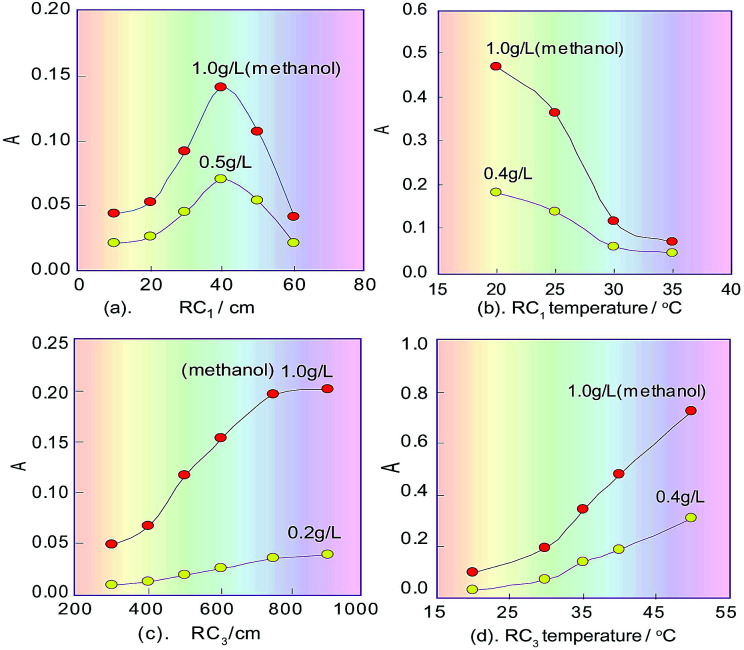
Effects of the lengths and temperatures of the reaction coils in the FIA system on the response signals. (a) Examination of the effect of the first coil length; (b) the temperature effect for the first reaction coil; (c) the effect of the third coil length; (d) the temperature effect of the third reaction coil.

In the spectrophotometric flow-injection analysis system, the sensitivity of methanol content analysis is mainly controlled by the residence time of the methanol sample plug which is injected into the system, and the temperature within RC_1_ when methanol is oxidised into formaldehyde. Because the residence time is proportional to the length of RC_1_, the effect of the length on the sensitivity was first examined under the selected conditions mentioned above, and the correlating results are shown in Fig. 4a. It can be seen that when the RC_1_ length was 40 cm, the product absorbance relating to methanol content had a maximum value, which proved that the process of KMnO_4_ oxidizing methanol to generate formaldehyde is a fast reaction. In the non-equilibrium flow system, if the RC_1_ length was too long, methanol in the sample plug would be excessively oxidized and changed into formate, which would lead to the decreasing of the subsequent product and therefore the sensitivity of the system. Of course, if the length was too short, the amount of formaldehyde formed by oxidizing methanol would be too small, which would lead to a reduced amount of the subsequent product and so a decrease in the sensitivity of the system. Therefore, the RC_1_ length was selected as 40 cm.

On the basis of the parameters optimised above, the temperature effect on KMnO_4_ oxidizing methanol was investigated by changing the RC_1_ temperature (KMnO_4_) in the range 20–40 °C. As can be seen in Fig. 4b, the sensitivity of our system to detect methanol decreased as the temperature of the KMnO_4_ solution increased. This phenomenon suggested that the reaction between KMnO_4_ and methanol is an exothermic reaction. Finally, the temperature of RC_1_ was selected to be room temperature (about 20 °C).

When the redundant KMnO_4_ flows into RC_2_, a fading reaction would take place with K_2_S_2_O_5_ (which was introduced from another line), so the RC_2_ length will affect the extent of fading of the KMnO_4_. If the RC_2_ length was too short, the redundant KMnO_4_ could not be completely faded, which would affect the next colour-producing reaction, but if the length was too long, it would increase the dilution of formaldehyde that is generated in the sample plug, and so decrease the analysis speed. Therefore, the effect of the RC_2_ length on the product absorbance in the reaction system was examined. The experiment results showed that there was a maximum response signal at 100 cm for the RC_2_ length.

The sensitivity of the system for methanol content analysis secondly depends on the generation time of formaldehyde, and the temperature within RC_3_ where the colour-producing reaction occurs. Therefore, the effect of the RC_3_ temperature and length, which is proportional to the residence time of formaldehyde, was evaluated under the designated conditions mentioned above. The results are shown in Fig. 4c, d. As can be seen from Fig. 4c, the absorbance change of the coloured product increased in the range 300–700 cm, and when the length exceeded 700 cm, the absorbance no longer changed. This implies that the colour-producing reaction between formaldehyde and FSS in RC_3_ is a slow reaction, so the RC_3_ length should not be too short. Fig. 4d indicates that the absorbance of the coloured product proportionally increased as the chromogenic reaction’s temperature increased in the range 20–50 °C, namely, sensitivity of the flow system increased with RC_3_’s temperature increase. This phenomenon suggests that the reaction between formaldehyde and FSS is an endothermic reaction. Considering the response range and the baseline noise of the detector, the temperature of RC_3_ was set at 50 °C with a thermostat.

### Effect of sampling volume in the FIA system

**Fig. 5:**
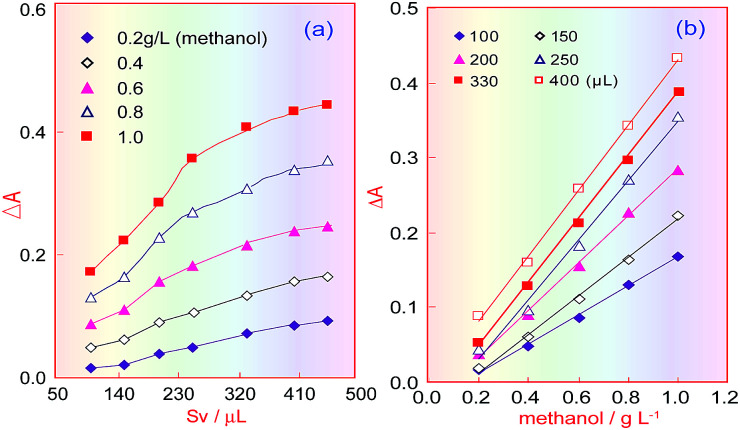
Effects of the sample volume on determining methanol content in the FIA system. (a) Influence of different sampling volumes on absorbance; (b) correlation curves between absorbance and methanol concentration under different sampling volumes.

Under the selected conditions mentioned above, the effect of the sample volume (*S*_v_) was investigated in the range 100–450 μL. Fig. 5a shows that when the sampling volume increased, the product absorbance increased under different methanol concentrations, and that this increasing trend became slow when the sample volume increased above 330 μL. Fig. 5b indicates that the absorbance of the coloured product proportionally increased with increasing methanol concentration, but that when the sample volume was larger than 330 μL, only the blank absorbance (intercept) increased, and so the sensitivity (the curve slope) did not increase. Therefore, the sampling volume was selected as 330 μL.

### Temperature effects for FSS/ethanol and FSS/methanol reactions

**Fig. 6:**
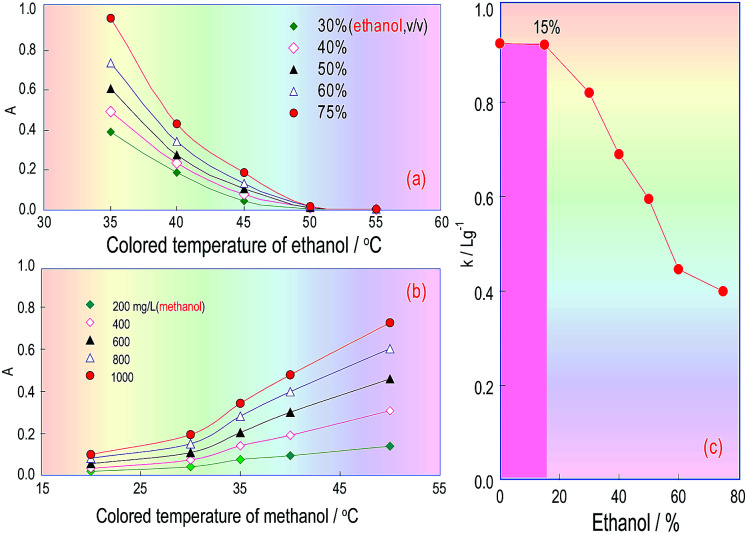
Temperature effects of the chromogenic reagent on the determination of methanol and ethanol amounts in the same system. (a) Influences of the chromogenic reagent’s temperature on ethanol content determination; (b) the effect of temperature on methanol content determination; (c) the effect of ethanol content in an ethanol/methanol mixture solution on the methanol calibration curve’s slope.

Because ethanol and the absorbance of the coloured product are correlated in the KMnO_4_/K_2_S_2_O_5_/FSS reaction system, FSS’s temperature effect on the reaction between FSS and acetaldehyde formed by ethanol in RC_3_ was also investigated. The ethanol standard used for the test samples was injected into the FIA system, and the absorbance curves of the coloured product associated with the FSS/acetaldehyde (ethanol) reaction were obtained under different temperatures. Fig. 6a indicates that when the FSS temperature increased from 35 °C to 50 °C, the absorbance of the coloured product quickly declined, and when the temperature was greater than 50 °C, the absorbance was close to zero. This showed that the coloured product relating to FSS/acetaldehyde (ethanol) is unstable and decomposes easily at high temperature. As a consequence, we can conclude that the reaction between acetaldehyde and FSS is fast and exothermic, and can quickly occur at room temperature.

The temperature effect of the formaldehyde/FSS reaction in RC_3_ was also investigated under the same conditions, using the methanol standard for the test samples. As can be seen in Fig. 6b, the curve’s changing trend is similar to that in Fig. 4d, and so this further confirms that the formaldehyde/FSS reaction is slow and endothermic. Besides, it was seen with the naked eye that the product colour in the formaldehyde/FSS reaction was stable at high temperature and did not fade for a long time. Thus the temperature effect of formaldehyde is opposite to that of acetaldehyde in the same reaction system. This suggested that the ethanol interference in methanol analysis for wine can be eliminated by elevating the RC_3_ temperature and so the distillation pretreatment of the wine sample assay can be avoided. If so, the analysis time for methanol will be significantly reduced.

Accordingly, after the temperature of RC_3_ was set at 50 °C and the other FIA pipeline was set at room temperature (about 20 °C), the effect of the temperature on the absorbance of the coloured product from the ethanol/methanol mixing samples was estimated. The obtained results are shown in Fig. 6c, in which the abscissa denotes the ethanol concentration and the ordinate denotes the calibration curve slopes (*k*: L g^−1^) for the methanol standard solutions which contained ethanol at different concentrations. This curve reflects the extent of the impact of different ethanol amounts in wines on the methanol quantitation. Fig. 6c indicates that if the ethanol content in the methanol standards was less than 15%, the slope of the methanol calibration did not change, namely, as the ethanol content was less than or equal to 15%, it would not interfere with the methanol determination. If the content was over 15%, the slope of the methanol calibration started to observably decrease, namely, the ethanol content in the methanol standards began to interfere with the methanol quantification. The ethanol content in red wines is generally in the range 8–15%, so when the RC_3_ temperature in the FIA system is controlled at 50 °C, the interference coming from the ethanol content could be overcome, and the process of distilling the wine sample can be dispensed with.

### Effect of interfering substances on methanol measurement

In order to examine the influence on the method of other substances coexisting in wines, some constituents with different concentrations were proportionally added (1 + 1) to a 1000 mg L^−1^ methanol standard. These mixtures were then used as testing samples to detect using our system, and finally the tolerance value††Note: this was the ratio of the added content and the maximum inherent content of the constituent in the winesof these additives was judged according to the calculated recovery. The experiment results showed that K^+^(20), Na^+^(15), Mg^2+^(20), Mn^2+^(150), Ca^2+^(15), Br^−^(150), PO_4_^3−^(15), SO_4_^2−^(20), Cl^−^(20), citric acid (20), acetic acid (20), aldehyde (2), normal propyl alcohol (10), isobutyl alcohol (20), isoamyl alcohol (2), ethyl acetate (20), *etc*, did not interfere with the methanol determination. However, if the ethanol content was over 15% (v/v), it would have a positive effect, and if the amount of glycerol in the wines was more than twice the permissible value, it would also result in a positive effect. As well as this, some other coexisting micromolecular alcohols in wines (such as *n*-amyl alcohol, isoamylol, *etc*), did not interfere with the determination of methanol, because the relative amounts were much smaller than ethanol and methanol in the wines.

### Determination of methanol content in the samples

Five kinds of red wine were selected as representative samples, and their methanol content was detected under the above optimized conditions. To eliminate the effects of the chrominance and the reagent blank on the methanol content analysis, as well as the innate formaldehyde in these wines, the H_3_PO_4_ solution was first introduced into the FIA system through the three-way valve (V_2_) under systemic parameters. The other reagents were fixed in place, and the wine samples were injected into the carrier stream in turn to respectively get their blank absorbance (*A*_b_). Then, the 3-way valve was switched to another position to introduce the KMnO_4_/H_3_PO_4_ oxidant, and the same wine samples were again injected into the system to obtain the absorbance of their coloured products (*A*_m_). Finally, the methanol content of the wines was quantified by calculating the absorbance difference (Δ*A* = *A*_m_ − *A*_b_).

**Fig. 7:**
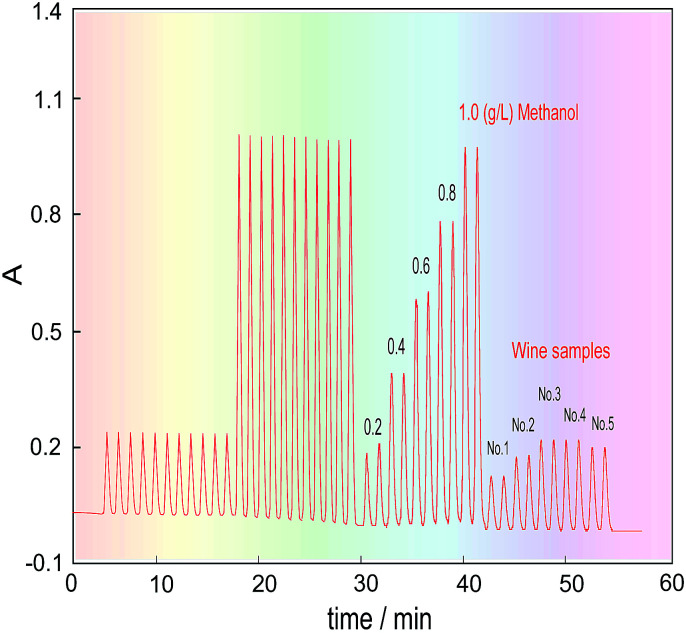
Real curves obtained by determining the methanol content in the wines.

The detected and calculated results are shown in Table 1, and the real detection curves are shown in Fig. 7. Due to the alcohol content in the wines, which is normally about 11.5–12.5%, the methanol standard was made using 12% ethanol as the solvent. The purpose of this is to make the background level of the methanol standard solution match with that of the wine samples.

**Table 1:** Direct determination of methanol content in wine using our system (*n* = 3)

No.	Samples (diluted 1 fold)	*A*_b_	*A*_m_	Δ*A*	Methanol con.^a^/mg L^−1^
1	Muscat	0.057	0.148 ± 0.001	0.09	180
2	Cabernet Sauvignon	0.136	0.198 ± 0.001	0.062	118
3	Ruby Cabernet	0.095	0.217 ± 0.002	0.121	249
4	Changyu Cabernet	0.123	0.222 ± 0.002	0.098	203
5	Gorelli	0.115	0.227 ± 0.002	0.112	228

a (1) Δ*A* = 1.09*c* − 0.0084 (*r* = 0.9997); (2) this comes from the data multiplied by the dilution multiple.

In order to estimate the accuracy of the determination results, a recovery test was carried out by adding a known amount of methanol into the wine samples. In the experiment, samples from different wines were divided into six portions which were separately added into the iso-volumetric methanol standards (0, 200, 400, 600, 400 and 1000 mg L^−1^). Then the absorbance of the coloured product from these mixtures was detected by the analytical system. The obtained recovery values in Table 2 are in the range 95–105%, which is satisfactory. This experiment suggests that our method is able to conduct accurate and direct determination of the methanol content in wines.

**Table 2:** Recovery tests of wine samples with the FIA system (*n* = 3)

Samples	Sample conc./mg L^−1^	Added conc.^a^/mg L^−1^	Determined conc./mg L^−1^	Recovered conc./mg L^−1^	Recovery/%
Changyu Cabernet	203 ± 4	200	203.0 ± 5	101.5	102
		400	311.1 ± 6	209.6	105
		600	398.2 ± 11	296.7	98.9
		800	497.3 ± 10	395.8	99.0
		1000	602.4 ± 8	500.9	100
Ruby Cabernet	249 ± 7	200	224.00 ± 5	99.5	100
		400	318.1 ± 6	193.6	96.8
		600	416.2 ± 3	291.7	97.2
		800	509.3 ± 3	384.8	96.2
		1000	614.4 ± 15	489.9	98.0
Muscat	180 ± 5	200	194.0 ± 3	104.0	104
		400	299.1 ± 3	209.1	105
		600	407.2 ± 6	317.2	106
		800	488.3 ± 6	398.3	99.5
		1000	578.4 ± 6	488.4	97.7
Cabernet Sauvignon	118 ± 5	200	159.1 ± 6	100.1	100
		400	248.5 ± 12	189.5	94.8
		600	356.9 ± 9	297.9	99.3
		800	438.2 ± 8	379.2	94.8
		1000	576.4 ± 16	517.4	103
Gorelli	228 ± 9	200	218.0 ± 6	104.0	104
		400	323.1 ± 11	209.1	105
		600	404.2 ± 1	290.2	96.7
		800	512.3 ± 9	398.3	99.6
		1000	617.4 ± 15	503.4	101

a The volumes of standard and sample solutions were mixed by one plus one.

### Contrast experiment

#### Manual colorimetric method

When the manual method^34^ was used for the methanol quantitation, the ethanol in the wines caused interference, so before the analysis, the wine samples were distilled according to the boiling-point difference between ethanol and methanol.

The same volume (5 mL) of the methanol standard and the pretreated wine sample was added into two separate 25 mL tubes, and a 2 mL mixture of the KMnO_4_ and H_3_PO_4_ solution was added to each tube. After heating the tubes for 10 min at 30 °C, 2 mL of the H_2_C_2_O_4_/H_2_SO_4_ mixture was added again into the two tubes. Once the tubes had cooled to room temperature, 5 mL of FSS was added to the tubes, and they were left to react at 30 °C for 30 min. Finally, the reaction solutions were detected using the spectrophotometer. The determined results are shown in Table 2.

#### Gas chromatography analysis

**Fig. 8:**
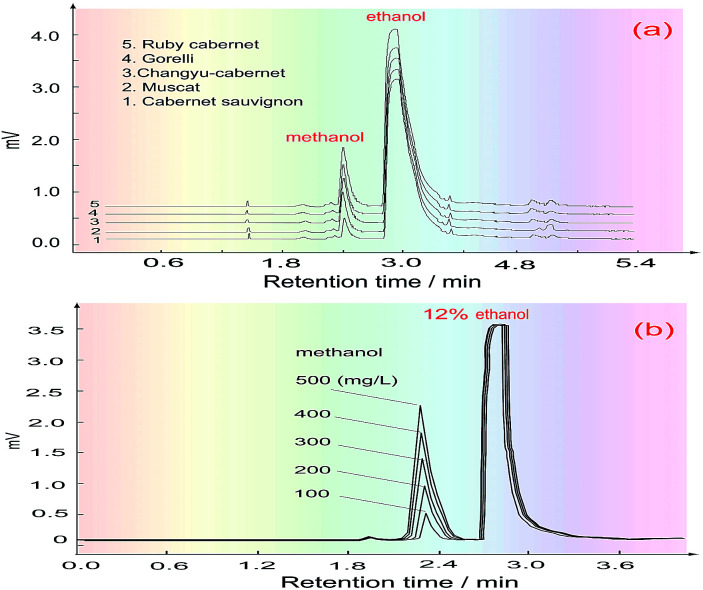
The GC peaks for determining the methanol content of the wines. (a) GC peaks of the wine samples; (b) GC peaks of the standard samples.

The gas chromatography conditions for methanol detection were as follows: a KB1701-type quartz capillary column (30 m × 0.32 mm × 0.50 mm) was used for separating the methanol and ethanol; nitrogen gas (15 mL min^−1^) was used as the carrier gas; hydrogen gas (30 mL min^−1^) and air (200 mL min^−1^) were respectively used as fuel and oxidant gases; the temperature of the detector and the gasification chamber was 220 °C; the oven temperature was 50 °C; the sampling volume was 1.0 μL. The real detection curves for the methanol content are shown in Fig. 8, and the related data are shown in Table 2. Obviously, the results obtained using our method are consistent with one of two reference methods.

## Conclusions

The optimized conditions for our system to determine the methanol content of wines were as follows: the concentration of KMnO_4_ was 20 g L^−1^; the concentration of FSS was 3 g L^−1^; the length of RC_1_ was 40 cm; the length of RC_2_ was 100 cm; the length of RC_3_ was 700 cm (I.D.: 0.8 mm); the temperature of RC_3_ was 50 °C; the temperature of RC_1_ and RC_2_ was about 20 °C; the sample volume was 330 μL. The proposed method and system all possesses noteworthy advantages in the areas of automated operation, repeatability, test cost and sample pretreatment, and realized the direct and rapid determination for methanol in wines. Its determination result was consistent with the reference methods. Consequently, the method and system can serve as a supplementary standard for methanol content determination, and can also be used for online quality control of the winemaking process and the rapid determination of methanol content in the final wine product, as well as in low-alcohol drinks.

## Conflicts of interest

There are no conflicts to declare.

## Supplementary Material

## References

[cit1] Brown M. R., Ough C. S. (1982). Am. J. Enol. Vitic..

[cit2] Gnekow B., Ough C. S. (1979). Am. J. Enol. Vitic..

[cit3] Kumar V. , Abul K. and Aster J. C., Robbins Basic Pathology, Elsevier, 9th edn, 2013

[cit4] Kruse J. A. (1992). Intensive Care Med..

[cit5] Li Y. S., Qi J. N., Gao X. F. (2006). Liquor-making Sci. Technol..

[cit6] Bindler F., Voges E., Laugel P. (1998). Food Addit. Contam..

[cit7] GB/T15307, Wine, National Standards of the People’s Republic of China, 2006

[cit8] Feher J., Lengyel G., Lugasi A. (2007). Cent. Eur. J. Med..

[cit9] Garrigues J. M., Pérez-Ponce A., Garrigues S., Guardia M. D. L. (1997). Vib. Spectrosc..

[cit10] Wang M. L., Wang J. T., Choongy Y. M. (2004). J. Food Compos. Anal..

[cit11] Caruso R., Gambino G. L., Scordino M. (2011). Nat. Prod. Commun..

[cit12] Ai G., Sun T., Dong X. (2014). Rapid Commun. Mass Spectrom..

[cit13] Zhang Y. C., Lin N. B., Chai X. S., Li Z., Barnes G. D. (2015). Food Chem..

[cit14] Gülce H., Gülce A., Kavanoz M., Coşkun H., Yildiz A. (2002). Biosens. Bioelectron..

[cit15] Rotariu L., Bala C., Magearu V. (2004). Anal. Chim. Acta.

[cit16] Chen S. H., Wu H. L., Yen C. H., Wu S. M., Lin S. J., Kou H. S. (1998). J. Chromatogr. A.

[cit17] Kuo C. C., Wen Y. H., Wu S. S. (2003). Anal. Lett..

[cit18] Ruzsicka J. (2006). Trends Anal. Chem..

[cit19] Li Y. S. , and Gao X. F., Flow injection analysis and application for chemistry analysis, The Jilin People’s Publisher, China, 2002, ISBN 7-206-02728-8/R 37

[cit20] Gao X. F. , Ikebukuro K., Karube I. and Li Y. S., LRA, Wiley & Sons, 1997, vol. 9, pp. 69–79

[cit21] Araújo A. N., Couto C. M. C. M., Lima J. L. F. C. (1998). J. Agric. Food Chem..

[cit22] Li Y. S., Muo Y., Xie H. M. (2002). Anal. Chim. Acta.

[cit23] Li Y. S., Xing C. X., Yang L. L. (2005). Anal. Sci..

[cit24] Li Y. S., Zhao B., Sun X. H. (2009). Spectrosc. Spectral Anal.

[cit25] Li Y. S., Zhao B., Sun X. H. (2009). J. Sichuan Univ., Eng. Sci. Ed..

[cit26] Nanita S. C., Stry J. J., Pentz A. M., McClory J. P., May J. H. (2011). J. Agric. Food Chem..

[cit27] Li Y. S., Zhou L., Zhang H. S., Lai Z. Y., Gao X. F. (2012). Anal. Sci..

[cit28] Gao X. F., Ikebukuro K., Karube I., Li Y. S. (1997). Bunseki Kagaku.

[cit29] Gao X. F., Li Y. S., Karube I. (2001). Anal. Chim. Acta.

[cit30] Maria C. G. D., Manzano T., Duarte R., Alonso A. (1995). Anal. Chim. Acta.

[cit31] Almuzara C., Cos O., Baeza M., Gabriel D., Valero F. (2002). Biotechnol. Lett..

[cit32] Kelly J., Chapman S., Brereton P., Bertrand A., Guillou C., Wittkowski R. (1999). J. AOAC Int..

[cit33] Horwitz W. and Latimer G. W., Official methods of analysis of AOAC international, 18th edn, 2011

[cit34] GB/T15038, Analytical methods of wine and fruit wine, The National Standard of the People’s Republic of China, 2006

[cit35] Liu S. G., Liang Y. Z., Cao Z. Y. (2005). Chin. J. Anal. Lab..

[cit36] Yu B., Dai X. F., Zhang S., Chen Y. G. (2006). J. Shanghai Univ..

[cit37] Li Z. W., Ma H. B., Lu H. H., Tao G. H. (2008). Talanta.

[cit38] Zhang Z. Q., Yan H. T., Yue X. F. (2004). Microchim. Acta.

[cit39] Yue X. F., Zhang Z. Q. (2007). J. Anal. Chem..

[cit40] González-Rodríguez J., Pérez-Juan P., Castro M. L. D. (2002). Anal. Bioanal. Chem..

